# Comparing In-ear EOG for Eye-Movement Estimation With Eye-Tracking: Accuracy, Calibration, and Speech Comprehension

**DOI:** 10.3389/fnins.2022.873201

**Published:** 2022-06-30

**Authors:** Martin A. Skoglund, Martin Andersen, Martha M. Shiell, Gitte Keidser, Mike Lind Rank, Sergi Rotger-Griful

**Affiliations:** ^1^Division of Automatic Control, Department of Electrical Engineering, The Institute of Technology, Linköping University, Linkoping, Sweden; ^2^Eriksholm Research Centre, Part of Oticon A/S, Snekkersten, Denmark; ^3^T&W Engineering A/S, Allerød, Denmark; ^4^Department of Behavioral Sciences and Learning, Linneaus Centre Head, Linköping University, Linkoping, Sweden

**Keywords:** EOG, audio-visual, speech comprehension, eye-tracking, in-ear EEG, hearing impairment

## Abstract

This presentation details and evaluates a method for estimating the attended speaker during a two-person conversation by means of in-ear electro-oculography (EOG). Twenty-five hearing-impaired participants were fitted with molds equipped with EOG electrodes (in-ear EOG) and wore eye-tracking glasses while watching a video of two life-size people in a dialog solving a Diapix task. The dialogue was directionally presented and together with background noise in the frontal hemisphere at 60 dB SPL. During three conditions of steering (none, in-ear EOG, conventional eye-tracking), participants' comprehension was periodically measured using multiple-choice questions. Based on eye movement detection by in-ear EOG or conventional eye-tracking, the estimated attended speaker was amplified by 6 dB. In the in-ear EOG condition, the estimate was based on one selected channel pair of electrodes out of 36 possible electrodes. A novel calibration procedure introducing three different metrics was used to select the measurement channel. The in-ear EOG attended speaker estimates were compared to those of the eye-tracker. Across participants, the mean accuracy of in-ear EOG estimation of the attended speaker was 68%, ranging from 50 to 89%. Based on offline simulation, it was established that higher scoring metrics obtained for a channel with the calibration procedure were significantly associated with better data quality. Results showed a statistically significant improvement in comprehension of about 10% in both steering conditions relative to the no-steering condition. Comprehension in the two steering conditions was not significantly different. Further, better comprehension obtained under the in-ear EOG condition was significantly correlated with more accurate estimation of the attended speaker. In conclusion, this study shows promising results in the use of in-ear EOG for visual attention estimation with potential for applicability in hearing assistive devices.

## 1. Introduction

There is a strong scientific and commercial trend toward developing more realistic assessment methods for the development of new technology. The main reason is the urge to obtain results that are applicable to real-life scenarios. In hearing sciences, and related fields, this concept is referred to as ecological validity (Keidser et al., 2020). Experiments conducted in less controlled environments that better represent the real world may introduce higher variability and unexpected effects in data, making analysis and interpretation of data more challenging. Sensing technologies can improve scene, situation, context, and intention awareness (Mehra et al., 2020; Slaney et al., 2020), and are promising tools for use in research with less experimental control. In hearing research, there is particularly a growing interest in sensing eye movement that is used as a metric to identify what sound (e.g., speech source) the listener is attending to. This information can be used to identify the best signal processing strategy to apply in hearing devices to optimize hearing in hearing-impaired people. For example, in Best et al. (2017) and Roverud et al. (2017), a highly directional beamformer array was steered by the participants' visual attention using a conventional eye-tracker and multi-speaker conversational stimuli. Their results showed improved performance when the speaker was fixed in a single location, but suggest that it is harder to improve speech intelligibility when the target speech location switches in an unpredictable fashion. Also using a conventional eye-tracker, eye-behavior in dyadic conversation was studied in Hadley et al. (2019), where it was found that increasing noise led to more focus on the speaker's mouth, stressing that eye movement may be a good metric for identifying the speaker attended to in a multi-speaker scenario and thus steering hearing device algorithms. But for real-life applications, the use of conventional eye-tracking devices is inconvenient.

Portable electroencephalography (EEG) is a promising technology that has recently received considerable attention within the fields of, for example, neuroscience and psychology. The low price point, ease-of-use, and portability makes portable EEG systems attractive for integration into sensing platforms that can support experiments in real-life scenarios. EEG systems enable attended sound sources to be decoded from cortical brain responses (O'Sullivan et al., 2014; Fuglsang et al., 2017) with realistic sound stimuli, but these systems typically require several seconds of data and are therefore not yet an option for real-time steering applications. In contrast to selective attention, eye movements can be measured with only temple and forehead electrodes *via* electrooculography (EOG). Given this, it is a good candidate for online applications requiring fast estimates (see e.g., Manabe and Fukumoto, 2006; Favre-Felix et al., 2018; Chen et al., 2019; Belkhiria and Peysakhovich, 2021; Gunawardane et al., 2021; Kastrati et al., 2021). With an across-ear referenced setup, EOG in the transverse plane can be measured with in-ear electrodes. This is particularly of interest for integration in hearing devices (Fiedler et al., 2016) when combined with dry-electrode solutions (Kappel and Kidmose, 2015, 2018; Kappel et al., 2018) that can be used without conductive gel and can easily be used in real-life situations. In Favre-Félix et al. (2017) and Hládek et al. (2018), fixation angles of eye-gaze were estimated in real-time with in-ear EOG, showing great potential for hearing device steering applications. Furthermore, auditory attention estimation (Grimm et al., 2018), using direction-of-arrival and EOG with the purpose of estimating probabilistic sound-source localization, has been evaluated for beamforming in hearing aids. This evaluation demonstrated that EOG can successfully assist in analyzing the soundscape.

In previous work conducted at our laboratory (Favre-Félix et al., 2019), an LED-light was placed on each loudspeaker and was used to indicate which loudspeaker the user should steer their attention to, similar to the setup described in Pomper and Chait (2017). The attended loudspeaker was amplified based on the absolute eye gaze angle in the horizontal plane as estimated using EOG, inertial sensors, and magnetometers. The absolute gaze angle was, however, difficult to estimate. The EOG signal is heavily affected by the noise that is associated with, for example, DC drift and muscle activity, which generates large variability in the results. Other potential drawbacks with that setup were that the participants sometimes scanned the scene to detect when LEDs switched, and the use of LED-lights and lack of other visual cues were not particularly representative of real-life scenarios. Furthermore, the study only used eleven hearing-impaired (HI) participants and speech intelligibility, which was measured using the DAT speech corpus (Bo Nielsen et al., 2014), was not analyzed and reported.

To fully take advantage of sensing technology in future experiments, new assessment methods are needed (Carlile and Keidser, 2020; Lunner et al., 2020). To this end, the increased interest in the more ecologically-valid hearing research outcomes has resulted in more frequent use of audio-visual stimuli and the development of more challenging speech paradigms in hearing research, refer to e.g., Llorach et al. (2018), for an overview of advanced setups introduced for that purpose. The impact of visual cues, and the corresponding eye-gaze behavior, on speech comprehension in complex listening conditions has not yet been fully investigated. For this purpose, an audio-visual test paradigm that targets speech comprehension of a natural dialog has recently been developed at our laboratory (see Cabella, 2021 for an application of this paradigm).

The main objective of the current study was to further develop and validate the application of in-ear EOG for attended speaker estimation in a realistic listening situation. For this, we compared in-ear EOG estimation to that obtained with a conventional eye-tracker. Furthermore, given that in-ear EOG signal quality can vary greatly between electrodes par and over time, we proposed and evaluated a calibration method that presents three metrics for visual inspection to evaluate EOG signal quality in order to extract the best channel from 36 possibilities. The method was assessed against the eye-tracking ground truth reference for estimating the attended speaker (i.e., conventional eye-tracking). Finally, we assessed whether in-ear EOG eye-steering improves speech comprehension for HI participants in this realistic listening situation. Performance with EOG steering was compared to performance with eye-steering *via* a conventional eye-tracking device and no eye-steering.

## 2. Materials and Methods

### 2.1. Participants

Twenty-seven HI test participants were recruited from the Eriksholm clinic test pool based on the following selection criteria:

- Test participants should not have been previously exposed to audio-visual stimuli.
- Test participants should be native Danish speakers.
- Test participants should not use eye glasses, unless their sight deficit was sufficiently negligible to not impair their vision of the experiment stimulus, or they could replace their eye glasses with contact lenses during testing.
- Test participants should have mild to moderate hearing loss with a maximum of 60 dB at all frequencies. This constraint could be relaxed to 70 dB at the highest frequency i.e.,8 kHz.
- Test participants should not have large hearing-level asymmetries:
  - Asymmetry at each frequency should not exceed 20 dB.
  - Average asymmetry across frequencies should not exceed 10 dB.

**Figure 1:**
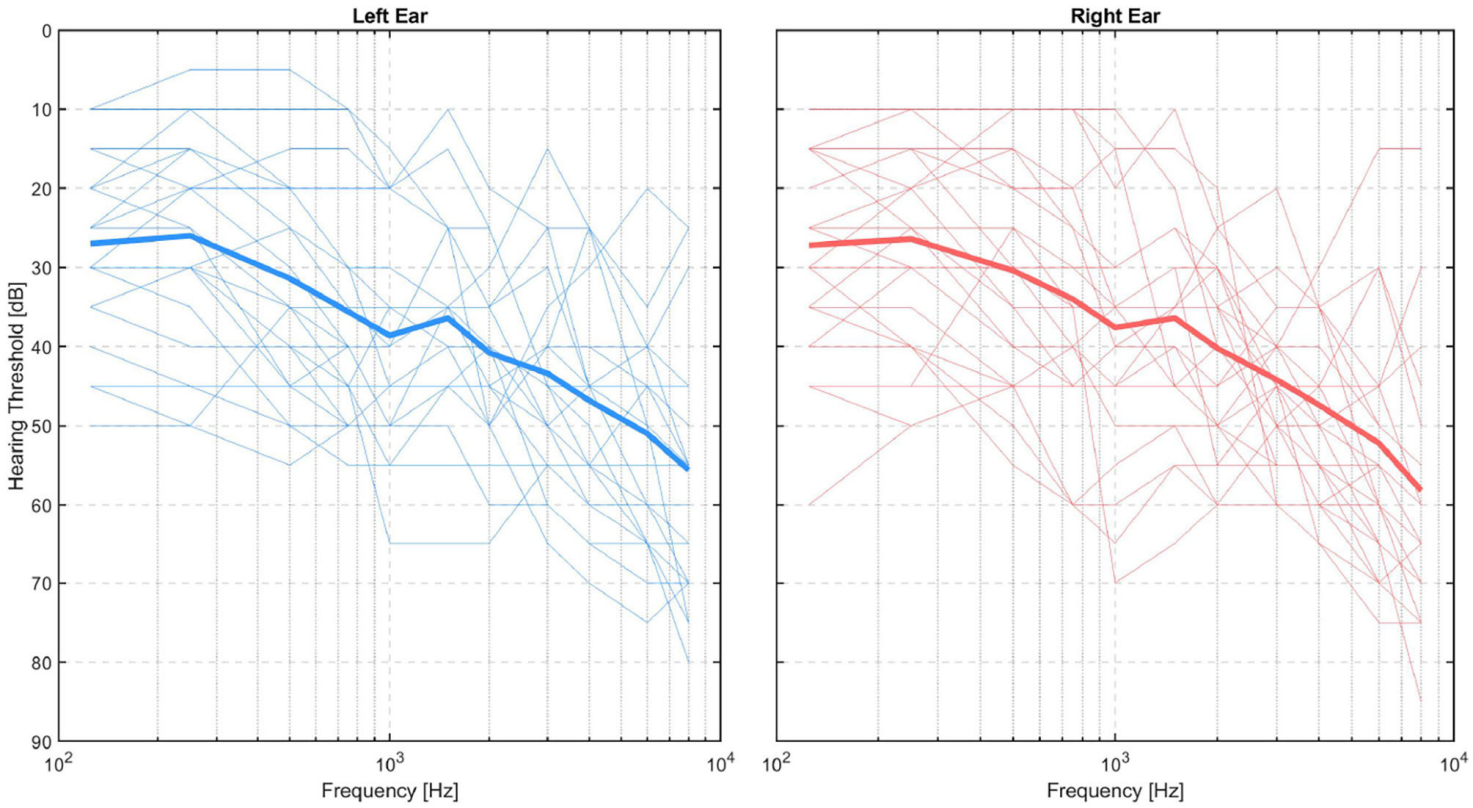
Audiograms of test persons shown separately for left (blue lines) and right (red lines) ears, with the group mean per ear represented by thick lines.

All participants signed a consent form and the experiment was approved by The Ethics Committee of the Capital Region in Denmark (H-20030989). Out of the 27 participants, one was excluded due to unavailability to complete all experiment sessions, and one was excluded due to the inability to perform the task. For each of the remaining 25 participants, we collected data for 6 different conditions presented in separate blocks, making a total of 25*6=150 data blocks. Of these data blocks, four were discarded due to different issues, e.g., missing data or missing triggers, leading to a total of 146 valid data blocks. The gender distribution of the final sample was 11 females and 14 males, and the ages were distributed with a mean of μ = 69.5 *years* and a SD of σ = 8 *years*. Thresholds were measured at each audiometric frequency from 125 to 8,000 Hz. The audiograms of all participants who are included in the results are shown in Figure 1.

### 2.2. Technical Setup

**Figure 2:**
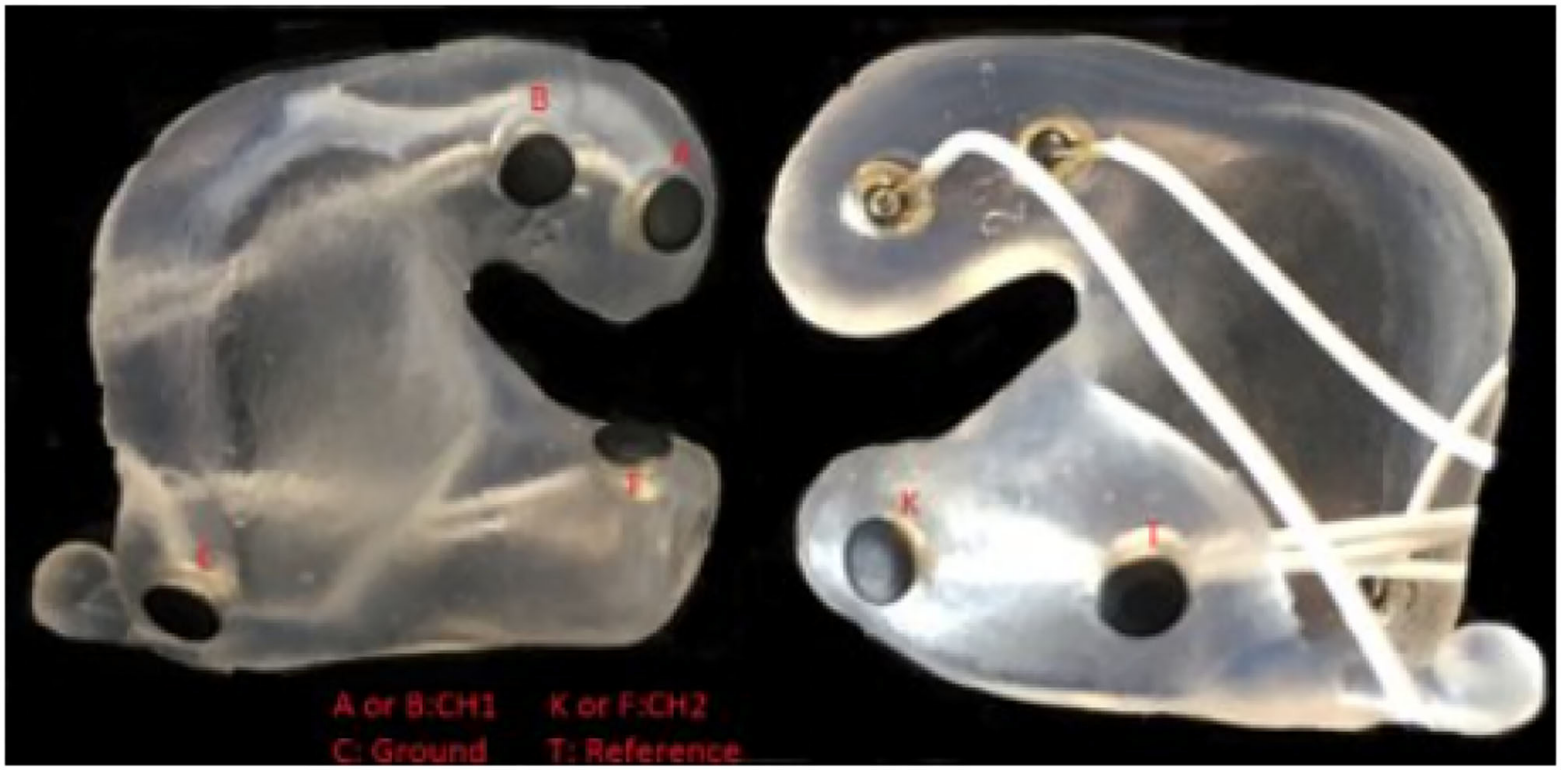
Example of a soft silicone mold equipped with EOG electrodes.

**Figure 3:**
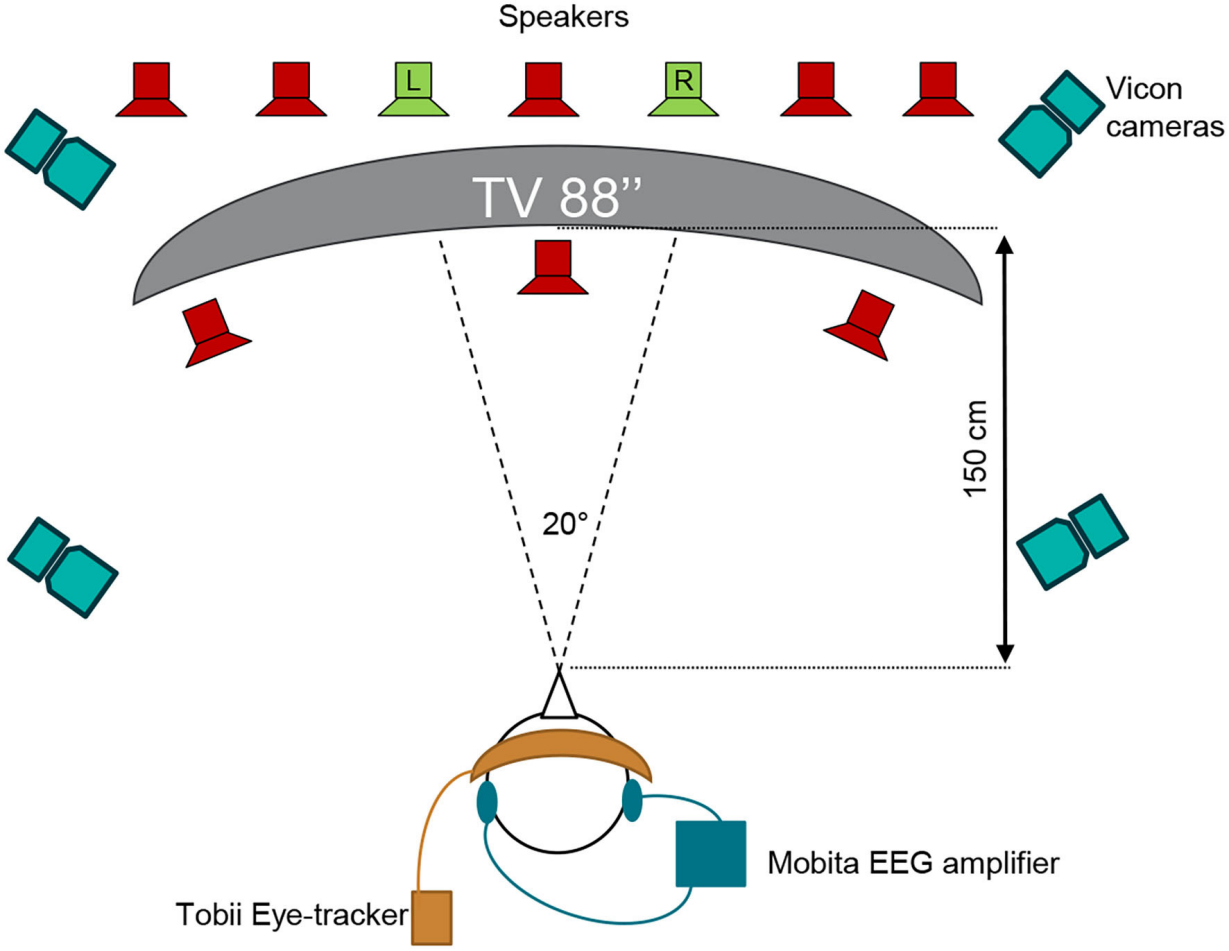
Schematic overview of the test setup with Vicon motion capture cameras, Tobii Pro eye-tracking Glasses 2 used to measure gaze, the Mobita EEG amplifier used to record in-ear EOG signals, and the 88 inches 4K TV used to present life-sized persons in the video stimuli. The two green loudspeakers present the conversation material and are spatially aligned with the two speakers on the TV while the eight red loudspeakers present babble noise.

**Figure 4:**
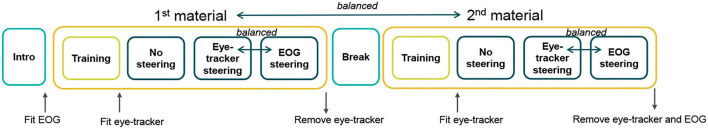
Schematic overview of the procedure used for each test person.

The test was executed at the Eriksholm Research Centre (ERH) in June 2021. Prior to the experiment, the participants had ear impressions made, from which silicone molds with embedded in-ear electrodes were produced, as shown in Figure 2 for an example. The technical setup of the experiment is illustrated in Figure 3. Participants were seated in a comfortable chair in front of an 88 inch curved TV. Participants were fitted with EOG ear molds made from individual ear impressions equipped with 6 dry contact electrodes, which was connected to a 32 channel Mobita EEG amplifier from TMSi, which sampled at 500 Hz as described in Kappel et al. (2018); Tobii Pro Glasses 2 mobile eye-tracking device, which sampled at 50 Hz, and included reflective markers that were recorded *via* a Vicon motion capture system, which sampled at 100 Hz; and two behind-the-ear (BTE) devices for recording audio, accelerations, rotational velocity, and magnetic field. The BTE data was not used in the current study. The Vicon motion tracker was used to monitor potential movement of the test participants' heads. MATLAB 2019a was used to present the audio-visual stimuli, see Section 2.3, on the TV. Target speech was directionally presented from two loudspeakers, and babble noise in the frontal hemisphere was presented from eight loudspeakers, further explained in Section 2.3, and simultaneously presented from 10 loudspeakers situated in front of the participant *via* both a Fireface UCX soundcard from RME and a Hammerfall DSP Multiface II. The two loudspeakers in green in Figure 3 presented the target speech and were roughly spatially aligned with the position of the speakers on the screen. For the comprehension task, questions and response options were shown on the screen, and participants answered the questions using a Bluetooth keyboard. Data from all capturing devices (Tobii, EOG, and Vicon) were synchronized with a signal delivered through the sound card. The one-point calibration, as part of the Tobii glasses setup, was done before each recording block if it was deemed necessary. A desktop computer and a laptop were used in this complex setup. The desktop computer recorded Tobii and Vicon data and executed the stimulus MATLAB script. The EOG data was collected on the laptop which also executed the eye-steering algorithms using MATLAB 2016b. To enable communication of the attended speaker between the laptop and the desktop computer, a Maya USB+ 44 soundcard sent audio signals between the computers. Figure 3 illustrates the test set-up while Figure 4 describes the test flow.

The hearing loss of the test participants was compensated on the stimulus side based on the audiogram for a better ear. The compensation was computed with the CAMEQ formula for linear hearing aids (Moore and Glasberg, 1998), with the CAMEQ output extrapolated from 6 to 9 kHz using a cubic spline. This way of compensating for hearing loss can lead to a loud environment and that is why the hearing loss of the test participants was restricted to the mild-to-moderate range. The experimenter, who was present in the room during testing, wore ear protection.

### 2.3. Audio-Visual Stimuli Targeting Speech Comprehension

**Figure 5:**
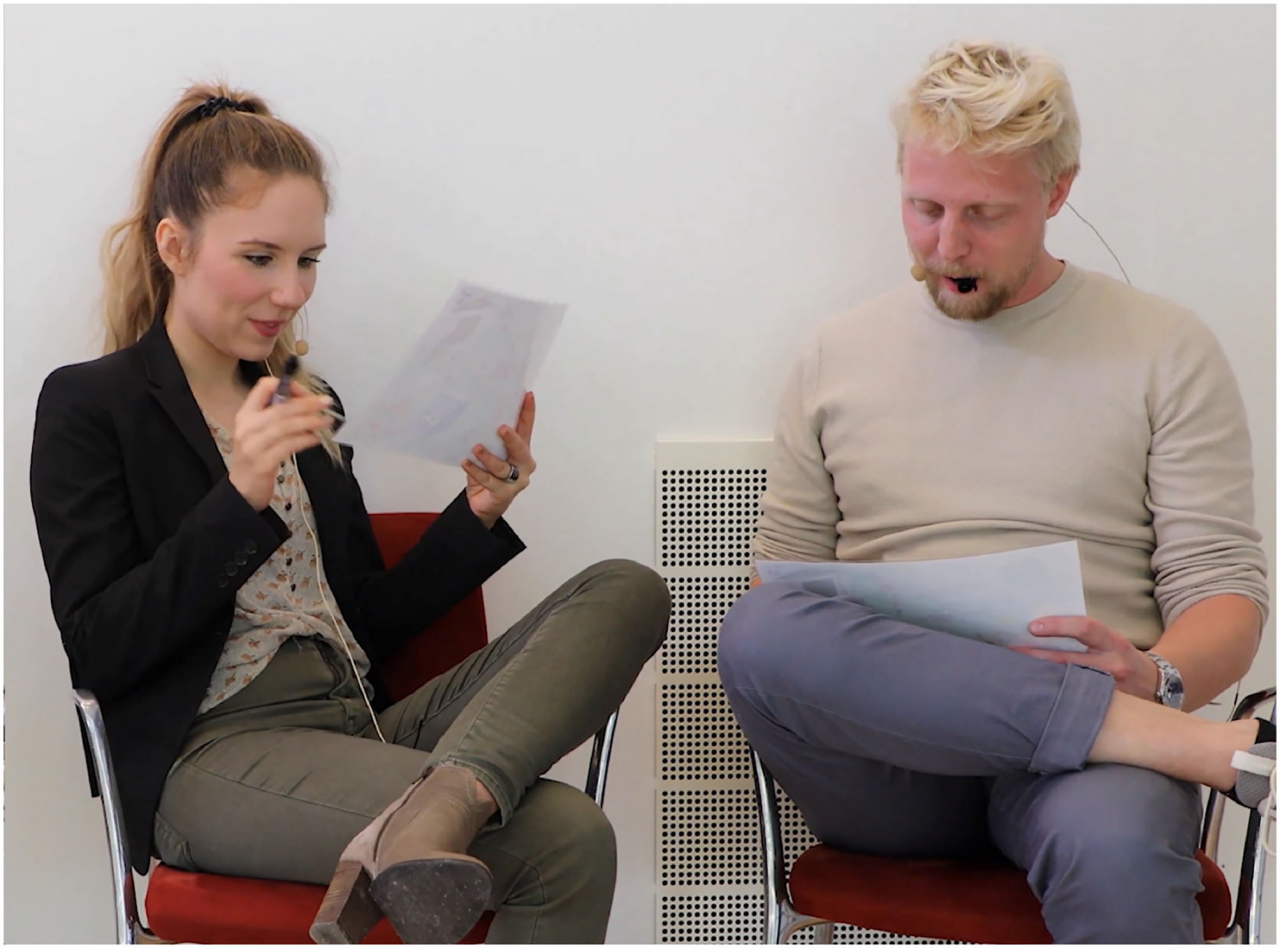
Still photo from the audio-visual stimuli with two actors in dialog solving a Diapix task (Baker and Hazan, 2011).

The stimuli and tasks were derived from Cabella (2021) and consisted of in-house HD video recordings of two speakers engaged in an unscripted conversation in Danish, refer to the example screenshot in Figure 5. Speakers were fitted with hands-free microphones and their speech was recorded on two separate audio channels. Two different sets of speakers were used, with pairs have taken either from four paid actors, herein referred to as the ACT material or four volunteers recruited among Eriksholm Research Centre staff, herein referred to as the ERH material. The speakers' conversation consisted of them solving a Diapix task (Baker and Hazan, 2011) wherein they verbally compared two similar drawings to find differences between them. The conversations were clipped into trials ranging between 10 and 39 s long, with each clip ending after the speakers uncovered a difference. These clips were preceded by a central fixation cross on a black screen for 3 s. The ACT material was used to generate a babble-noise that was subsequently used with both materials.

In order to assess how well the listener understood the conversation that was presented in the stimulus, a multiple-choice question with three response options followed each clip. In this question, the participant was asked to identify what the speakers identified as the difference between their drawings. The options consisted of broad categories that described reoccurring differences in the test materials, such that individual response options could be used for multiple different trials. Because these response options were not identical to the words spoken in the conversation, this testing strategy pushed the listener to understand the meaning of the conversation rather than merely recognize words. Note that for the ACT materials, the questions and stimuli were previously used in an experiment with 11 HI listeners (Cabella, 2021). For the ERH materials, the questions were piloted with a small number of normal-hearing and hearing-impaired participants prior to the experiment.

A total of 97 different trials were used in the experiment, with 67 and 30 from each of the ACT and ERH materials, respectively. The ACT material was divided into a practice block of 13 trials, used to familiarize the participants with the task, and 3 blocks of 18 trials each. The ERH material was divided into a practice block of 6 trials, and 3 blocks of 8 trials. Each condition was tested with a separate block of trials from each material, and the same block was used for every participant. This allowed for comparison between participants within a block, for the purpose of assessing the success of the proposed calibration procedure. Since the no-steering condition is the most difficult for the participants, it was always presented first for each type of material, thereby reducing the probability of participants giving up from fatigue. This choice was considered in favor of complete block randomization with the assumption that further learning effects after the initial practice blocks are negligible. The trial order was randomized within each condition for each participant, and the order of the steering conditions was counterbalanced between participants.

### 2.4. Steering Conditions

Each participant carried out the comprehension task under three different conditions:

- No-support: Constant SNR set to 0 dB for the full experiment. Sound levels for the attended speaker, unattended speaker, and noise were all set to 60 dB SPL.
- EOG steering: Sound levels were the same as for the no-support condition, but a 6 dB gain was provided to the estimated attended speaker computed from EOG data.
- Eye-tracker steering: Sound levels were the same as for the no-support condition, but a 6 dB gain was provided to the estimated attended speaker computed from eye tracking data.

Before testing a material condition, the participants completed a training block to familiarize themselves with the task and stimuli. In the training block, the SNR was 10 dB, with the target speech presented at 60 dB SPL and the babble noise at 50 dB SPL.

### 2.5. Eye-Tracker Reference and Steering

**Figure 6:**
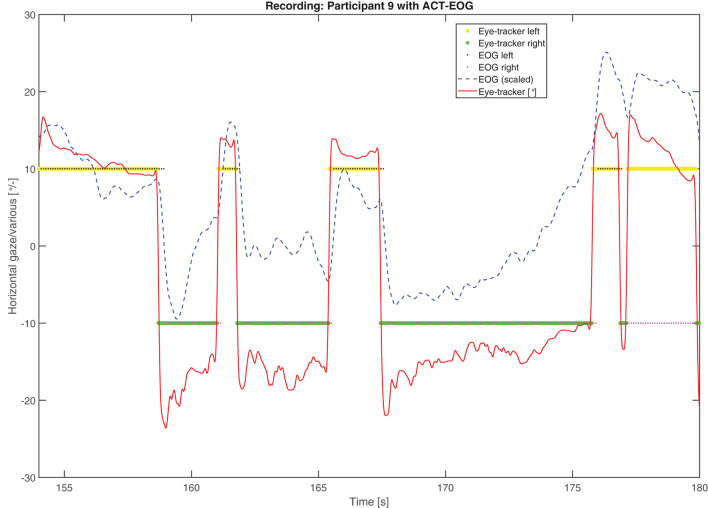
An example of four channels showing the highest correlation (Corr). Reference signal in solid red, EOG signal in green, assumed saccade events in red overlaid on the green line, and detected saccades represented by black dots.

Eye-tracker gaze data in the horizontal azimuth was used to determine which of the two speakers the participant attended at each time point, see yellow asterisk (left speaker) and green asterisk (right speaker) in Figure 6 for an example. For this, attention to a speaker was defined as gaze within that speaker's respective hemifield. Subsequently, these data were used as a ground-truth reference for performance in comparison to the EOG steering.

### 2.6. EOG Signals and Steering

Electro-oculography signals are proportional to eye deflection and are produced by the cornea-retinal potential in the human eye. Here, the potentials were measured with across-ear referenced, dry contact, and in-ear electrodes and were most sensitive to eye-movements in the horizontal azimuth. The dry contact electrodes, also used in Favre-Félix et al. (2019) and described in e.g., Kappel and Kidmose (2015), Kappel and Kidmose (2018), and Kappel et al. (2018), were based on a titanium substrate coated with iridium dioxide and mechanically designed to be embedded into a soft earpiece. There were 6 electrodes per ear and since the Mobita EEG amplifier uses a common mode reference, there were a total of 36 single different possible channel combinations.

#### 2.6.1. Saccade Detection and Attended Speaker Estimation

The electro-oculography steering algorithm estimated which of the two speakers the user attended at any point in time. Due to the skin-electrode interface (Huigen et al., 2002), the signals were vulnerable to low-frequency drift in the same order as the EOG potential, which challenges the calculation of an absolute gaze angle. This drift may be caused by the pre-amplifier, the contact potential changes, or changes in the EOG potential. However, saccades (fast eye-movements), such as those that a listener makes when switching their gaze between the two speakers, generate high-frequency responses that can be distinguished from the drift. We worked with the assumption that saccades were only generated by a switch in the listener's gaze between the speakers and not a switch elsewhere in the scene, and furthermore that the eyes remained fixated (on a speaker) when not performing a saccade. Hence, attended speaker estimation hinged upon accurate and robust saccade detection, which we based on the derivative of the EOG signal. Prior to obtaining the derivative, a second-order Butterworth filter with passband 0.1–1 Hz was used to remove part of the baseline drift and most of the electronic high-frequency noise, such as EEG-based measurement, while keeping sufficient saccade information for analysis. Saccade detection required the signal to conform to the following criteria:

- The derivative of the signal was bounded by a lower and an upper threshold of 0.1 mVs^−^ and 10 mVs^−^, respectively.
- The duration of a saccade was bounded by a lower and an upper duration threshold of 0.2 and 0.9 s, respectively.
- The magnitude of a saccade was calculated based on the absolute amplitude change, and absolute amplitude changes smaller than 15 mV were removed.
- Saccades with the wrong direction, e.g., a saccade to the left when the left speaker is already attended, were excluded.

The criteria listed above were refined during extensive testing with this specific setup in order to optimize attended speaker selection while mitigating disturbances. Note that with this procedure, there was no need for an absolute mapping between EOG and horizontal gaze location, as only the hemifield separation was considered, i.e., left or right relative to the test person.

### 2.7. Pre-Block EOG Channel Selection

**Figure 7:**
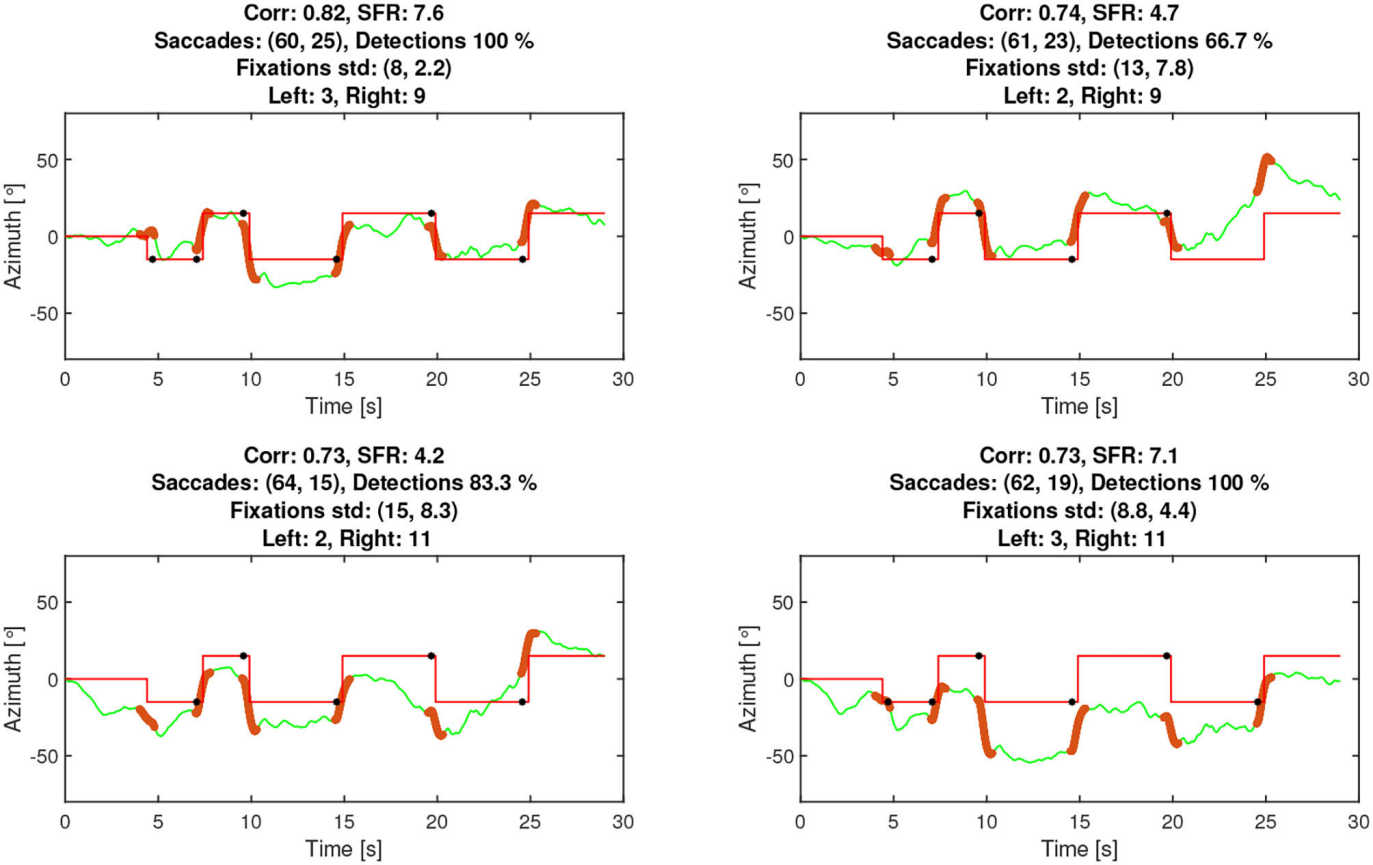
Example of attended speaker estimation data from a trial with participant TP1743. The solid red line represents gaze in degrees from the eye-tracker, the dashed blue line is the in-ear EOG signal, which is scaled for illustration purposes, the yellow and green asterisks are the eye-tracker-attended speakers while the black and magenta small dots are the in-ear EOG-attended speakers. Values below and above 0 deg. refer to left- and right-hand speakers, respectively.

To select the best of 36 possible channels for estimating the attended speaker, a calibration method was developed. Before each block, a calibration sequence was used consisting of a red dot that the participant was instructed to follow with their gaze. The dot moved between two horizontal positions on the screen in a pre-determined sequence. An example output of the calibration, as visible to the experimenter, can be seen in Figure 7. The positions of the dot approximated the speakers' locations at ±10° in the visual material. This allows various metrics to be computed based on all channel combinations, which were then used to select the best channel. The metrics computed were as follows:

- The correlation between the EOG and the reference signals. The higher the positive correlation, the better the channel.
- The proportion of correctly detected saccades using the EOG steering algorithm. The target value is 100%, corresponding to 6 out of 6, where the first saccade at 5 s is small, see Figure 7.
- The saccade-to-Fixation-Ratio (SFR) is the average saccade amplitude divided by the average standard deviation of the fixation. The higher the SFR, the better the channel.

The rationale for the SFR metric was that a high saccade amplitude corresponds to a better channel and that the signal should not fluctuate substantially during fixations, as it is expected to be a stationary mode. Note that SFR is also sufficiently simple to compute online and could potentially be used to monitor signal quality. The calibration output, as exemplified in Figure 7, was visually inspected by the experimenter to judge which channels to use. The two experimenters reported that using a combination of the plots and the metrics to select channels provided good support for finding channels and/or spotting errors.

## 3. Results

### 3.1. Attended Speaker Accuracy

**Figure 8:**
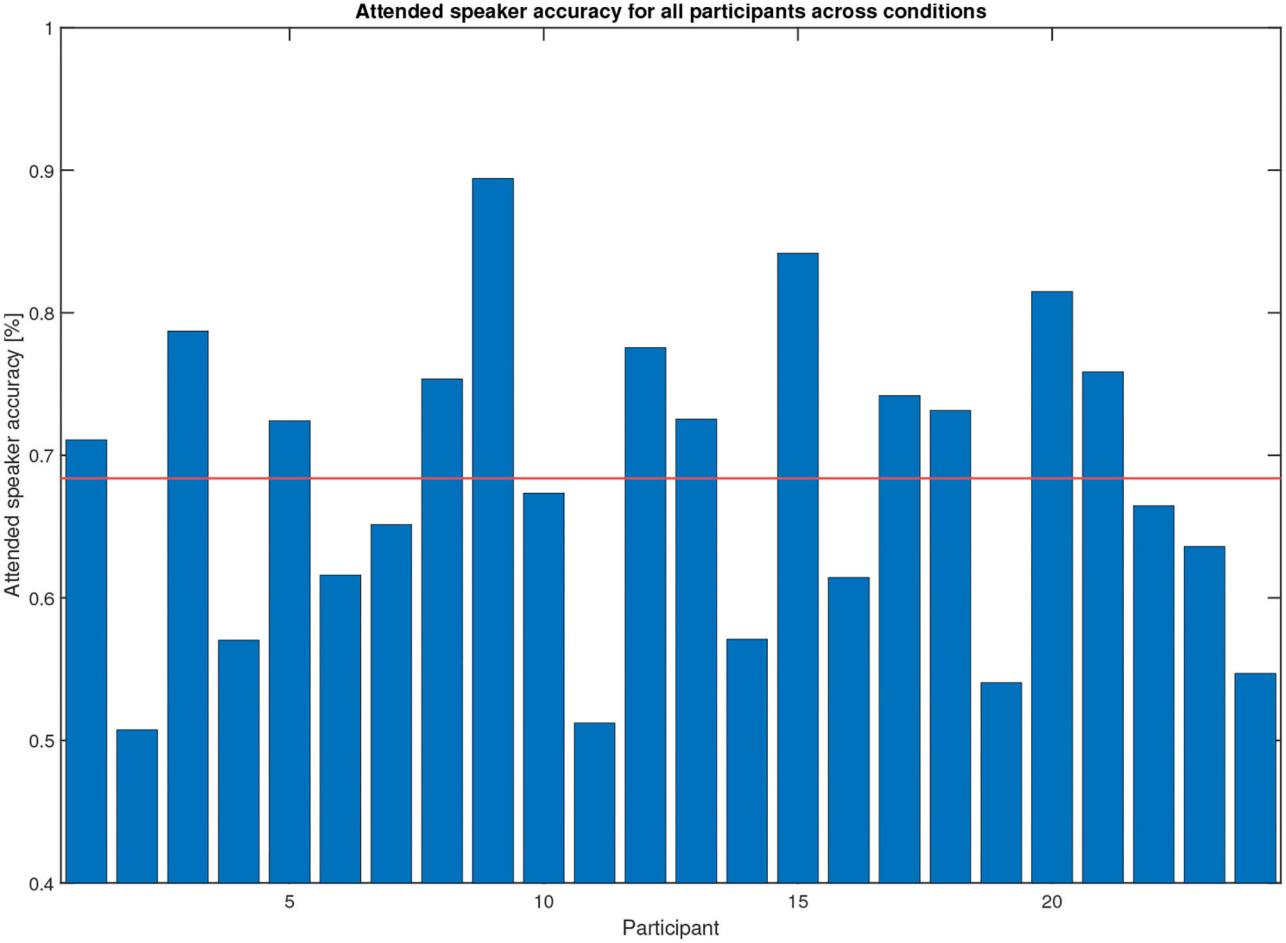
Attended speaker true accuracy across all conditions for the 25 participants (only 24 shown due to missing reference data for one participant). The mean of the true accuracy across participants is 68%. These data were computed during the experiment.

**Figure 9:**
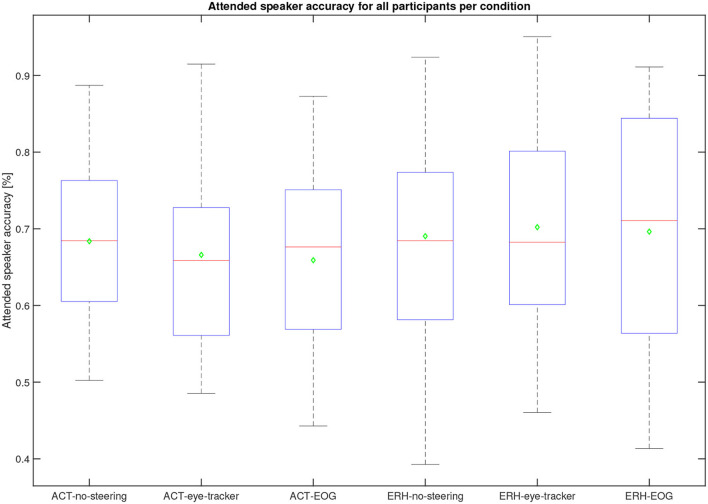
Average speaker accuracy across 22 participants (two were omitted due to missing data for one condition or more) for the different conditions. The median is the red line, the mean is a green diamond, the box represents the 25 and 75*th* percentile, the whiskers extend to the extreme values, and the red crosses represent individual outliers. These data were computed during the experiment. No significant difference is found between any of the conditions and materials.

The steering algorithm computed the attended speaker based on in-ear EOG as a time series, see the example in Figure 6, shown by black and magenta dots representing left and right speakers, respectively. The accuracy metric computes which percentage of time the in-ear EOG-estimated attended speaker matched the eye-tracker reference. A value of 100% means that the EOG data matched eye-tracker data perfectly, while the chance level is 50%. Note that the in-ear EOG attended speaker is initialized in the algorithm such that the first seconds of the trial may not reflect algorithm performance but chance, hence the first 3 s were removed in the accuracy measure for each trial. A scoring function was used to evaluate the in-ear EOG attended speaker accuracy based on the eye-tracker-attended speaker. Eye-tracker data within 2° with respect to zero azimuth was considered to be inconclusive and was omitted from the score. The attended speaker accuracy from the experiments across all conditions and materials for all participants is shown in Figure 8. One participant out of the 25 was omitted from this analysis as it had missing data in one or more conditions. The mean accuracy was 68%, the highest accuracy was almost 90%, and the worst was nearly down to chance level. In Figure 9, the estimated attended speaker accuracy is shown per condition and material, averaged across all participants. The accuracies per condition and material passed a Lilliefors test of normality. A one-way ANOVA did not support that there were any differences in average attended speaker accuracy between any of the six condition blocks [*F*_(5, 126)_ = 0.37, *p* = 0.87].

### 3.2. Calibration Evaluation

Since the calibration procedure code was developed for both online and offline use, it was possible to calculate the accuracies for all 36 electrode combinations using the recorded data. This allowed for an evaluation of the different strategies of selecting electrodes and assessing selection effectiveness during the experiment. The simulated attended speaker accuracy was computed using four different scenarios that may lead to the selection of different EOG channels. The scenarios were as follows:

- True accuracy: The channels that were selected by the experimenter using the calibration procedure.
- Best accuracy: The channel resulting in the highest accuracy among all 36 without considering the calibration.
- Corr accuracy: The best electrode combination out of all 36 as suggested by ranking the correlation estimated by the calibration procedure prior to each block.
- SFR accuracy: The best electrode combination out of all 36 as suggested by ranking the SFR score estimated by calibration procedure prior to each block.

**Figure 10:**
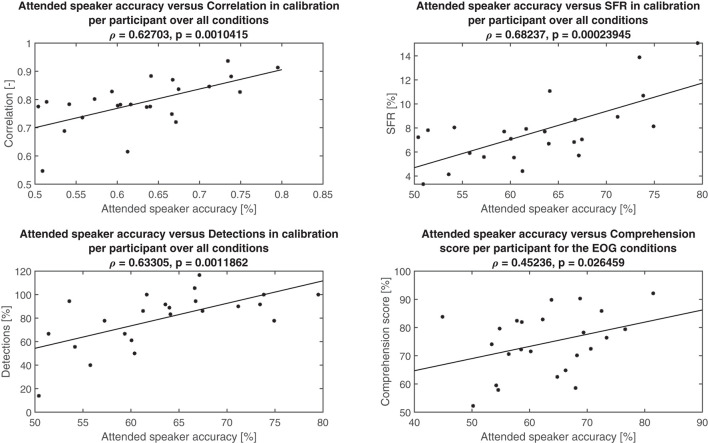
Attended speaker accuracy vs. various metrics per participant together with the least-squares line fit in solid, Pearson's correlation (ρ), and the corresponding *p*-value. Top left: Correlation metric from calibration procedure. Top right: SFR metric from the calibration procedure. Bottom left: Detection metric from calibration procedure. Note that one outlier showing unreasonable 300% detections was removed. Bottom right: Comprehension score in the EOG conditions.

Due to the technical setup and uncertainties in sampling rates, simulations with the recorded data did not align with the experimental data. For the purpose of this analysis, however, this was not of concern. Rather, it is the relative difference in attended speaker accuracy based on channel selection for the different scenarios that are of interest. The simulated True accuracy gave a mean of 63% compared to the accuracy mean from the online computation at 68%, resulting in a 5% difference in mean accuracy. The other three methods (Best, Corr, and SFR) produced means of 69, 59, and 62%, respectively. On this basis, we conclude that the experimenters selected effective channels with guidance from the calibration metrics. In Figure 10, the three calibration metrics, namely correlation, SFR, and detection (number of agreeable saccades), are plotted vs. the attended speaker accuracy per test participant, averaged across the conditions. All three metrics were related to attended speaker accuracy with correlations over 0.6. In the bottom right-hand plot, the average comprehension scores obtained in the in-ear EOG conditions per participant are shown. These were also positively correlated with attended speaker accuracy (ρ = 0.45236, *p* = 0.026459), such that comprehension scores improved as the EOG steering became more similar to the eye-tracker steering.

### 3.3. Speech Comprehension

**Figure 11:**
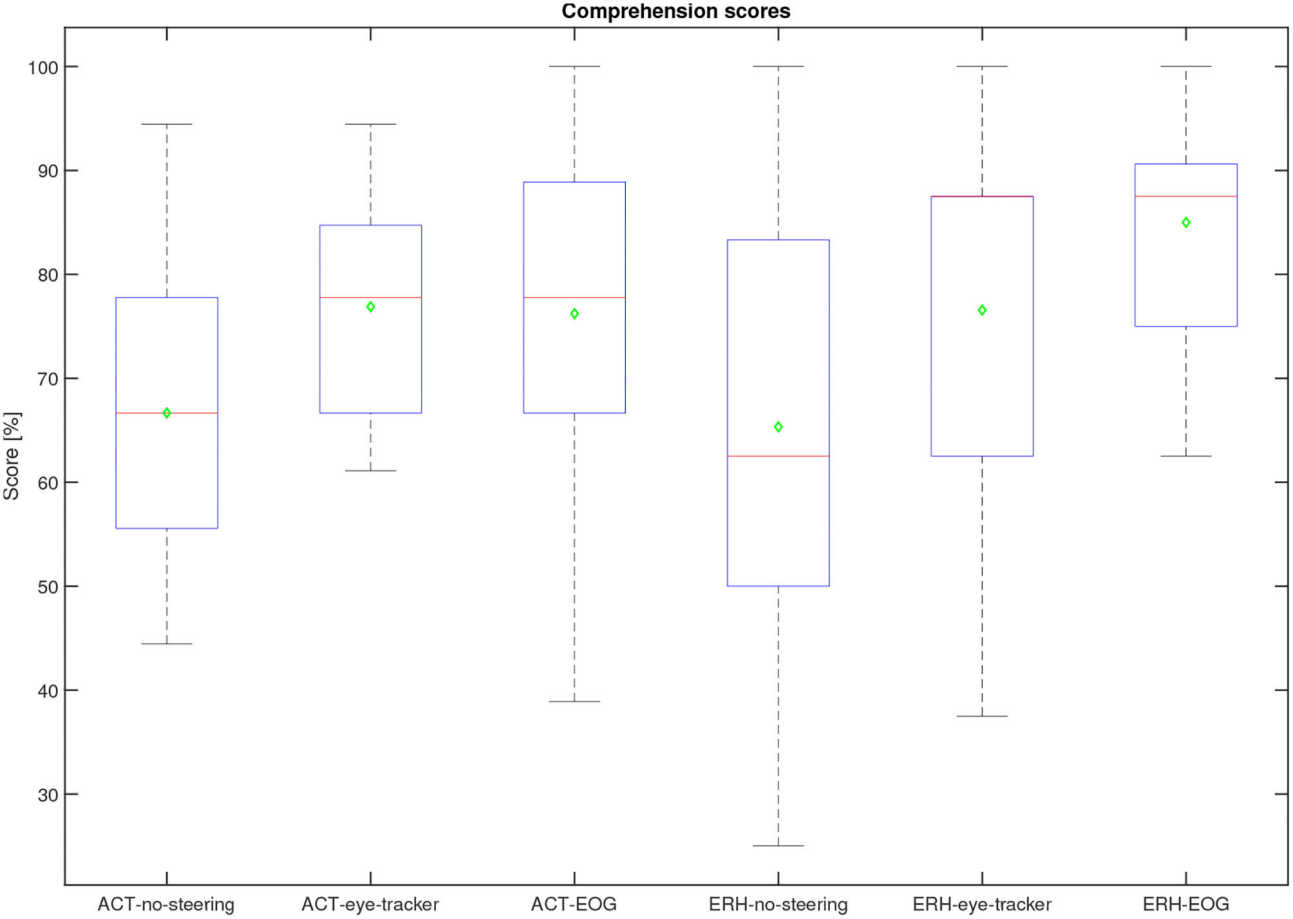
Comprehension performance scores for all participants per condition. The median is the red line, the mean is a green diamond, the box represents the 25 and 75*th* percentile, the whiskers extend to the extreme values, and the red crosses represent individual outliers.

**Figure 12:**
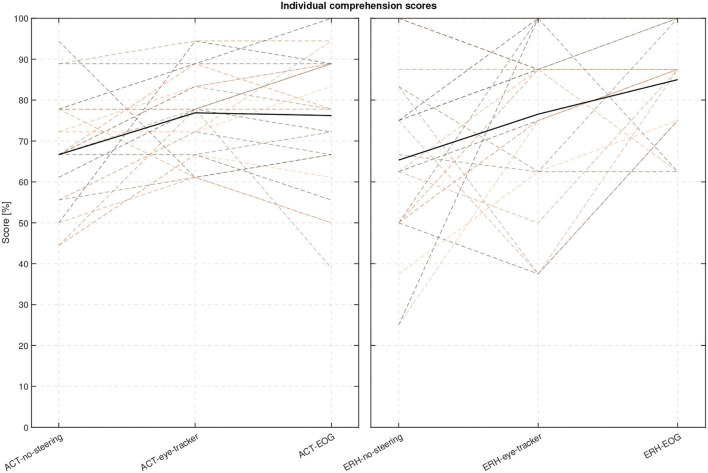
Individual comprehension performance scores for each participant and conditions with the mean scores shown as thick lines.

The overall performance scores in terms of speech comprehension in the audio-visual task are shown in Figure 11. As the task was a multiple-choice task with 3 possible answers, the data is binomial with the probability of success for a single trial being $\frac{1}{3}$. Based on the cumulative binomial distribution, the comprehension score chance level for the individual participant is 50% for the ERH material and 63% ACT material. Based on the work by Cabella (2021), that tested 7 hearing-impaired participants, the presentation SNR of 0 dB was expected to produce an average score of 60% for the no-support condition, which is sufficiently low to allow for potential improvement with the two steering conditions. As seen in Figure 6, this prediction was close to the actual outcome, with 66.7 and 65.3% correct for the ACT and ERH materials, respectively. The mean scores in the ACT-eye-tracker and ACT-EOG conditions were 76.8 and 76.2%, respectively. In Figure 12, the individual performance scores are shown together with the mean scores.

The following statistical analysis aimed to evaluate if there was a significant difference between the three steering conditions. One participant was omitted from this analysis as there were missing data in one condition, leaving 24 participants. A Lilliefors tests on each steering condition for each material show that not all data were normally distributed. Since only the steering conditions (no-support, eye-tracker, and EOG) were of interest here, and not the two types of materials, the comprehension scores were averaged across material types. The averaging of the scores was justified by a Mann-Whitney U-test showing no evidence of a significant difference in comprehension score between the two materials in each of the three test conditions: no-support (*U* = 597, *p* = 0.86), eye-tracker (*U* = 573, *p* = 0.76), and EOG (*U* = 528, *p* = 0.21). A Friedman test was conducted on the comprehension scores using the three conditions as independent variables. We found a significant difference in the comprehension scores between the different conditions (*p* = 3.5× 10^−5^, *Q* = 20.6). A *post-hoc* comparison of the comprehension scores in each condition was done with a Tukey-Kramer critical value test calculated at 5%. We found a significant difference between the no-support and both eye-tracker and EOG, with condition differences for no-support and eye-tracker (−0.88, *p* = 0.0056), no-support and EOG (−1.25, *p* = 2.89× 10^−5^). The condition difference between eye-tracker and EOG was not significant at (−0.38, *p* = 0.28). The ranks of eye-tracker and EOG conditions (2.17, 2.54) were both greater than no-support (1.29), supporting that the steering conditions have a positive effect on comprehension.

## 4. Discussion

As mentioned in the introduction, in-ear EOG has great potential for application in hearing devices to identify relevant speech among multiple speakers that the user wishes to attend to. The experimental setup used here, presenting an audio-visual dialogue, was intentionally limited in order to achieve robust and interpretable data and therefore does not represent the multitude of listening scenarios that hearing device users may be exposed to in everyday life. For example, contrary to the question-answer paradigm used in Best et al. (2017) and Roverud et al. (2017), where several speakers and directions were involved, the turn-taking in the current test, with only two speakers, is predictable. However, listening to dialog is a common real-life situation that can be seen as a building block for more complex multi-party conversations, and findings from this study should be helpful in refining steering technology for investigation in more complex settings.

Most of the participants had reasonable accuracies in the attended speaker EOG estimation as compared to the eye-tracker reference, and the overall mean was 68%. The accuracy of the attended talker estimation based on EOG was not significantly different across conditions or materials, and such a dependency was not expected either. The attended talker estimation based on saccade detection in EOG was robust and generalized well to the participants without the need for individualized parameters, which we believe is key for future applications. Beyond the scope of this paper but of interest for future endeavors on eye-movement analysis with EOG are comparisons of saccade detector designs and their performance; evaluation of other metrics, such as saccade rate and average fixation time with respect to SNR; and absolute angle estimation.

In line with previous work (Huigen et al., 2002; Hládek et al., 2018; Kappel et al., 2018; Favre-Félix et al., 2019), it was observed that some people have mostly distortion free EOG signals, leading to reliable saccade detections, while it can be extremely hard to find distortion free signals for others. Dependent on how well the ear-mold fits in the ear of the participant, a poor electrode-skin interface (Kappel et al., 2018) with weak signals and distortions may result. To support the experimenter in ensuring acceptable signals were obtained, a pre-block calibration procedure was developed utilizing three calibration metrics indicative of channel quality. The calibration procedure supported the finding of the most distortion free, but not necessarily the best, EOG signal to use in the experiments. All three calibration metrics were reasonable indicatives of attended speaker accuracy, with particularly the SFR calculation showing potential as a candidate for automated channel selection. This is because SFR is easily adapted for online signal quality monitoring without the need for reference data as saccades and fixations are already estimated in the algorithm. A restriction with the current setup is the across-ear referenced EOG which requires a wired connection between the ears, severely limiting hearing aid integration. Thus, future work should aim to introduce single ear EOG, which likely has worse SNR, further stressing the importance of calibration and signal quality monitoring procedure. For EOG methods relying on absolute angles, see e.g., Hládek et al. (2018) and Favre-Félix et al. (2019), the calibration is used to map the angles to voltage levels Manabe et al. (2013). These may vary between participants and require a reference for calibration. A potential alternative for hearing aid applications is to estimate speaker directions with binaural direction-of-arrival, see e.g., Braun et al. (2015), Zohourian et al. (2018), and Grimm et al. (2018), which is then used to calibrate the EOG.

Two steering conditions, one using conventional eye-tracking and the other in-ear EOG, provided significant comprehension improvement with reference to the no-steering condition. As long as participants paid attention to the active speaker in the dialog, an overall performance improvement in the steering conditions was expected due to the increase in SNR applied to the attended speaker. However, the improvement experienced with such steering systems will depend on several factors, such as the accuracy of attended speaker estimation, the complexity of the stimuli, and the signal processing applied to the attended sound. From previous analyses done in experiments that used the same material, more variation was found in the trials themselves than at different SNR levels (Cabella, 2021). Trials that were considered too easy or too difficult were only used for practice runs, and not used in the experiment. Even so, the remaining material variability resulted in difficulties finding an SNR associated with a 60% comprehension score for all participants in the no-support conditions. Part of this variability may be attributed to the different degrees of hearing loss presented across participants, even if it was compensated for. Ideally, the material variability would have a small influence on the average scores provided a sufficiently large number of participants were tested. Since, for logistic reasons, recruiting a large pool of participants was not possible for this study, it was instead decided to minimize the between-participant variance, and hence the variance within the test conditions, by fixating the trial material for each condition. There is therefore a confound on the material condition difficulty level that cannot be resolved in the analysis. Therefore, the comprehension results should be interpreted with care.

Although there was no significant difference between comprehension performance on the ACT and ERH material within each test condition, a slightly better overall performance was observed in the ERH material than in the ACT material, see Figure 11. There are two likely reasons for this. One is that the Eriksholm staff spoke more slowly and clearly than the actors, making the dialog easier to follow, and the other is that the questions developed for the ERH material were simpler and thus required less cognitive effort to answer. The babble noise which was used in both materials was generated from ACT material, which in general was a bit quieter than the ERH material. This means that for the same SNR condition (e.g., ACT-no-support and ERH-no-support), the SNR of the ERH material was slighly 0.83dB A higher than for the ACT material. This was known and accepted prior to the start of the data gathering. It remains for the future to better understand the differences and similarities between the materials, and also between individual trials, and then more systematically assess the effects they have on comprehension scores.

There was no significant difference between performance in the two steering conditions. It had been expected that the eye-tracker condition would perform better than the EOG condition because the EOG signals were not expected to be reasonably free of distortion for all participants, but the eye-tracker signals were. Apart from the possibility that the block of trials used for the in-ear EOG condition was easier to comprehend than those used for the eye-tracker condition, it is also possible that a certain amount of support was given even when the EOG steering did not react to saccades. This is because one speaker channel was always amplified by 6 dB, whether this was the channel for the speaker talking or not. This means that the participants could potentially pick up some keywords from the active speaker that was beneficial for comprehension, even if they did not directly attend to that speaker.

The non-significant difference in comprehension performance between the EOG and eye-tracker conditions suggest that there can be equal benefit from conventional eye-tracking and in-ear EOG steering. Thus, it should not be concluded that a lower attended speaker accuracy in the EOG condition is indicative of lesser comprehension improvement than in the eye-tracker condition, although differences in comprehension difficulties across the blocks used in the two conditions may have counteracted that. Nevertheless, the accuracy of estimating the attended speaker using in-ear EOG could never exceed that of the eye-tracker which is considered the ground truth reference. Due to the lack of a reference for the accuracy of eye-tracking, it cannot be deduced that this type of steering alone improves the comprehension score. However, as noted in Section 3.1, the comprehension score was improved in the EOG condition when the estimated attended speaker was more accurate. This can be taken as indirect support that when the accuracy of the EOG method is more similar to eye-tracker accuracy, then the speech comprehension is improved.

For future research, there are a few directions of particular interest. To fine-tune the experimental setup and to develop new stimuli that represent a variety of real-life communication situations, it is desirable to obtain a better general understanding of the validity of the comprehension paradigm introduced here; trial clip equivalence; and the significance of including visual components. Furthermore, developing a setup that allows us to better understand the interplay between steering and comprehension is desirable. It is likely that a simpler paradigm with more control of the task, for instance, speech intelligibility could be an option. These insights could guide the design for more efficient and intuitive attention switching algorithms and test paradigms.

## 5. Conclusion

A method for visual attention estimation using in-ear EOG was evaluated on hearing-impaired participants using an audio-visual dialog presented in noise. Particularly, a novel calibration procedure used to identify the strongest EOG signal available for estimating the attended speaker was investigated for accuracy against that obtained with a conventional eye-tracker. Comprehension performance with the two methods was also measured. The causal relationships found between the strength of various calibration metrics and greater attention estimation accuracy and better speech comprehension are highly encouraging and show great potential for utilizing in-ear EOG in hearing devices to steer signal processing strategies targeting the signal of interest.

## Data Availability Statement

The datasets generated and analyzed for this study can be obtained upon request.

## Ethics Statement

The studies involving human participants were reviewed and approved by Ethics Committee of the Capital Region in Denmark. The patients/participants provided their written informed consent to participate in this study. Written informed consent was obtained from the individual(s) for the publication of any potentially identifiable images or data included in this article.

## Author Contributions

The majority of manuscript preparation was done by MAS with assistance from all authors. MAS, SR-G, and MA developed the technical setup, software, and analyzed the data. SR-G and MA carried out the experiments and data collection. All authors contributed to the stimulus material development.

## Funding

This work was financially supported by the Swedish Research Council (Vetenskapsrådet, VR 2017-06092 Mekanismer och behandling vid åldersrelaterad hörselnedsättning).

## Conflict of Interest

MAS, MMS, GK, and SR-G were employed by Oticon A/S, and MA and MR were employed by T&W Engineering A/S. The reviewer VH declared a past co-authorship with one of the authors GK to the handling Editor.

## Publisher's Note

All claims expressed in this article are solely those of the authors and do not necessarily represent those of their affiliated organizations, or those of the publisher, the editors and the reviewers. Any product that may be evaluated in this article, or claim that may be made by its manufacturer, is not guaranteed or endorsed by the publisher.
